# Nasal eosinophil count and visual analog scale in patients with allergic rhinitis 

**DOI:** 10.5414/ALX02577E

**Published:** 2025-05-07

**Authors:** Giorgio Ciprandi, Irene Schiavetti

**Affiliations:** 1 Allergy Clinic, Department of Outpatients, Casa di Cura Villa Montallegro, and; 2 Health Science Department, University of Genoa, Genoa, Italy

**Keywords:** allergic rhinitis, visual analog scale, eosinophils, symptoms

## Abstract

None.

Sir, – Allergic rhinitis (AR) is a frequent disease characterized by typical symptoms and type 2 inflammation [1]. Notably, a patient-centered approach should drive AR patients’ management and self-management [2]. In this regard, the perception of nasal symptom severity is meaningful for properly managing AR patients. The visual analog scale (VAS) is the most reliable tool for assessing symptom perception [3]. In particular, it has been reported that VAS scores for perception of nasal obstruction correlated well with nasal airflow measured by rhinomanometry [4]. In addition, a VAS score > 5 may reflect uncontrolled AR [5]. As a result, patients use VAS in clinical studies, daily practice, and apps. 

As eosinophils are the leading inflammatory cells signaling allergic inflammation, the present study investigated whether nasal eosinophil count could correlate with VAS score for nasal symptom intensity in AR patients. For this purpose, nasal cytology could be fruitfully used. Nasal cytology is a standardized method that may be performed at the point of care [6]. 

This retrospective experience collected nasal cytology exams of AR patients who visited an outpatient clinic during the pollen season. 

The study included 100 symptomatic and untreated allergic patients (48 males, mean age 37.7 years) evaluated during the wall pellitory pollen season. Patients with comorbid asthma, chronic rhinosinusitis with nasal polyps, or non-allergic rhinitis were excluded. Nasal cytology and VAS assessment for intensity of all nasal symptoms (itching, sneezing, rhinorrhea, and obstruction) were performed as previously described in detail [4, 6]. Briefly, nasal cytology included: sampling, processing, and microscope reading. Sampling required the collection of cells from the surface of nasal mucosa by nasal scraping performed with a plastic device. Samples were collected from the middle portion of the inferior turbinate under anterior rhinoscopy in both nostrils. The sample obtained was immediately smeared on a glass slide and air-dried. The sample was stained using the common May-Grünwald-Giemsa method for 30 minutes. The stained sample was read at light microscopy at high magnification (× 1,000). Eosinophils were counted and reported as the mean number in 10 high power fields. 

The VAS value ranged from 0 = no symptom to 10 = the most intense symptoms. 

The best cutoff point of cell count able to discriminate between patients with VAS > 5 or < 5 was estimated on the basis of the receiver operator characteristic (ROC) curve analysis. Statistical significance was set at p < 0.05, and the analyses were performed using GraphPad Prism software. 

The results showed a strong positive relationship (Spearman’s rho = 0.86, p < 0.001) between VAS scores and nasal eosinophil counts (Figure 1A). 

The patients were stratified into two groups considering the VAS cutoff of 5, with those with a score > 5 of the VAS scale 0 – 10 being defined as suffering from uncontrolled AR (5 of the VAS scale 0 – 10). The patients with uncontrolled AR had higher eosinophil levels than other patients (Figure 1B). Interestingly, a ROC curve analysis through the Youden index showed that the eosinophil cutoff for discriminating uncontrolled AR patients was 7.5, with an area under the curve (AUC) of 0.96, sensitivity of 86.2%, and specificity of 97.1% (Figure 1C). 

These findings confirmed the primary role exerted by eosinophils in driving type 2 inflammation in AR. Moreover, the intensity of nasal eosinophil infiltrate correlated well with the perception of severe nasal symptoms assessed by VAS. Furthermore, a cutoff of 7.5 could identify AR patients with uncontrolled disease. 

Therefore, the present experience demonstrated that nasal eosinophil count measured by nasal cytology could integrate VAS assessment in patients with AR at the point of care. Moreover, the relevance of symptom assessment by VAS underscores the patient-centered approach in managing AR patients. 

## Ethical approval 

The Review Board of the Casa di Cura Villa Montallegro approved the procedure and all patients gave their informed consent. 

## Authors’ contributions 

GC: conceptualization, data collection, writing, and editing; IS: formal analysis. 

## Funding 

None. 

## Conflict of interest 

None. 

**Figure 1:**
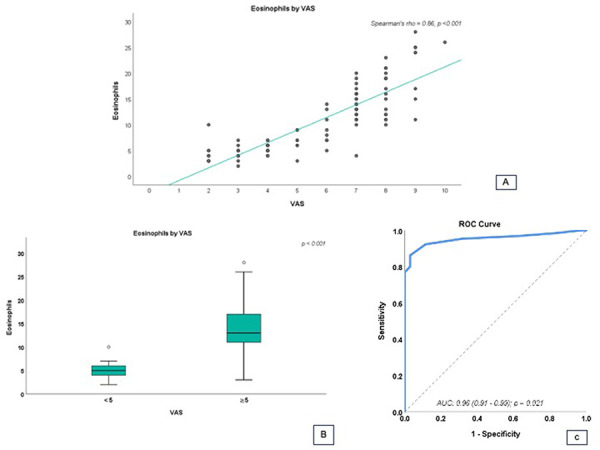
A: Relationship between nasal eosinophil count and visual analog scale (VAS) scores in patients with allergic rhinitis. B: Comparison between patients with a VAS score < 5 and patients with a VAS score > 5 considering the nasal eosinophil count. C: Receiver operating characteristic curve for defining the cutoff of nasal eosinophils for identifying patients with uncontrolled allergic rhinitis.
